# Changes in the Pre- and Postpandemic Unfinished Nursing Care Occurrence and Reasons as Perceived by Italian Nursing Students: A Secondary Analysis

**DOI:** 10.1155/jonm/8892363

**Published:** 2025-04-07

**Authors:** Stefania Chiappinotto, Tommaso Lupi, Aysun Bayram, Renzo Moreale, Luca Grassetti, Alvisa Palese

**Affiliations:** ^1^Department of Medicine, University of Udine, Udine, Italy; ^2^Faculty of Health Sciences, Karadeniz Technical University, Trabzon, Türkiye; ^3^Department of Economics and Statistics, University of Udine, Udine, Italy

**Keywords:** COVID-19, nursing students, pandemic, unfinished nursing care

## Abstract

Missed care, a phenomenon born in the United States more than 30 years ago and currently called unfinished nursing care (UNC), has been compared in pre- and postpandemic to detect changes in the trends as perceived by nurses. However, no studies have compared the perceptions of nursing students before and after these challenging times. The aim of this study was to compare pre- and postpandemic UNC occurrence and the reasons for it as perceived by Italian nursing students during their clinical rotations. A secondary analysis of data collected in 2018 (prepandemic) and 2023 (postpandemic) was conducted and here reported according to STROBE guidelines. The Unfinished Nursing Care Survey for Students (UNCS4S), measuring 22 expected interventions (from 22 [*never*] to 110 [*always left unfinished*]) and 18 possible reasons for it (from 18 [*nonsignificant*] to 90 [*very significant*]), was administered. The UNCS4S total scores of 231 (prepandemic) and 352 (postpandemic) students were 39.80 (CI 95% 37.06–42.54) and 50.89 (CI 95% 47.66–54.12), respectively (*p* ≤ 0.001). No significant differences between groups emerged for reasons (47.91, CI 95% 45.10–50.71 vs. 45.92, CI 95% 43.91–47.93, respectively; *p* = 0.257). Postpandemic students perceived a higher occurrence of UNC but with similar reasons to those reported before the pandemic.

**Summary**

• Postpandemic students perceived a higher occurrence of unfinished nursing care in units attended for their clinical education than prepandemic students.
• While some interventions are left unfinished first and others later, homogeneously, some have changed their order in the postpandemic, suggesting new prioritisation patterns.
• Reasons for unfinished nursing care perceived by students are in line with those reported in prepandemic times.

## 1. Introduction

Missed nursing care and tasks left undone research was established in the United States nearly 30 years ago and recently harmonised under an overarching term, unfinished nursing care (UNC), expressing the care required by patients but either not delivered or delayed [1]. Data regarding the occurrence of UNC suggest an increased occurrence during the pandemic, which continued after its conclusion due to the challenges faced by healthcare systems. The extreme pressure on healthcare facilities and the limited nursing capacity to provide the required care have been considered potentially increasing UNC and, consequently, adverse outcomes for patients [2], nurses [3] and organisations [4]. This trend has continued also after the pandemic, with a great crisis of most health services [5, 6] affecting the overall quality of care delivered [7].

To date, four reviews have summarised the body of knowledge produced during these challenging times. The first systematic review included 25 primary studies [8] documenting the intrapandemic UNC occurrence and the order of interventions missed (e.g., oral care), the reasons for which were substantially like those reported by nurses in prepandemic times. The second systematic review compared UNC between COVID-19 and non-COVID-19 patients, including only data from five intrapandemic hospital-based cross-sectional studies; it documented higher UNC occurrence among COVID-19 patients in the first wave, less compared to non–COVID-19 patients in the second wave, and other contrasting findings [9]. The third review, including 10 studies, revealed a shift in UNC compared to prepandemic data, with an emphasis on nursing care tasks vital for patients with COVID-19. The insufficient nursing workforce was confirmed as the primary UNC reason [10]. The fourth integrative review available focused only on reasons for UNC, including one cross-sectional and three intrapandemic qualitative studies, which reported new factors that were found to affect unfinished care at the system (e.g., new healthcare system priorities), unit (e.g., ineffective work processes) and patient levels (e.g., increased needs) [11].

Overall, the pandemic has challenged the preexisting frailty of the National Health Service, producing extreme working conditions [11]. However, substantial homogeneity in UNC occurrence has been reported with some differences in the reasons for it, suggesting two lines of interpretation: On the one hand, nurses have effectively faced the situation, ensuring the quality of care required despite the unprecedented increased needs; on the other hand, there are some limitations in the UNC measurements that have been conducted to date [12]. All pre- and intrapandemic studies have been conducted by collecting nurses' perceptions; however, their perceptions may have been biased due to the normalisation of UNC in daily practice and the tendency to underreport issues—for example, because of the implications for moral distress [13].

Some national documents (e.g., [14]) have suggested considering nursing students as a source of accurate data regarding issues in the quality of care. Although limitations and ethical implications have already been discussed in relation to their involvement in such evaluations [12], students have a ‘fresh pair of eyes' capable of perceiving UNC [15]. During their clinical rotations, they gain experience in different units, which allows them to detect differences; moreover, they can detect gaps between the acquired knowledge of how nursing care should be delivered and what is actually delivered. However, despite their potential relevance, only six qualitative [16–21] and two quantitative studies [22, 23] from the perspective of nursing students have been published to date in the field of UNC [12]. Moreover, no studies have detected changes by comparing pre- and postpandemic nursing students' perceptions regarding UNC occurrence and the reasons for it. Involving them may give value to the student's perspective and may integrate data collected from nurses to fully inform on the quality of the care delivered at the bedside. Therefore, this study was intended to expand the knowledge in the field and, ultimately, to gain a comprehensive picture of trends in UNC occurrences and reasons before and after the pandemic, as a proxy indicator of the quality of care delivered. Specifically, the primary aim was to compare differences, if any, in UNC interventions and the reasons for them as perceived by nursing students in the pre- and postpandemic periods; the secondary aim was to detect any differences, if any, in the order of interventions left unfinished between groups.

## 2. Methods

### 2.1. Study Design

A secondary analysis was conducted by considering data collected in two surveys performed in 2018 (hereinafter, prepandemic group) and in 2023 (hereinafter, postpandemic group given that data were collected after the end of the pandemic) [24]. Methodologically, the secondary analysis of existing databases allows (a) a cost-efficient way to maximise the use of data already available to address a new research question; (b) in a context where researchers have a comprehensive understanding of the strengths and weaknesses of the dataset, given that they were responsible for both primary surveys for the databases considered; and (c) an accurate comparison, given that variables in the two datasets were homogeneous in their operational definitions and data collection processes [25]. The methods and findings are here reported according to Strengthening the Reporting of Observational Studies in Epidemiology Statement for observational studies (Supporting Table 1) [26].

### 2.2. Setting and Population

The University of Udine (Italy) was approached and agreed to participate. The main features of the nursing program were stable over the years: The duration of the nursing degree course was three years and required around 1500 and 1800 h of theoretical and clinical education, respectively. Some changes were introduced to the nursing degree course between 2018 and 2023 due to the pandemic, such as online theoretical education and limited clinical placements [27]. Specifically, students belonging to the postpandemic group attended online/classroom lessons with fewer clinical placements, given the restrictions imposed by hospitals.

In both surveys, only students (a) attending the last week of their clinical rotation and (b) willing to participate in the study were eligible. Students attending the clinical internship for fewer than seven days were excluded as not being in the best position to report UNC, along with those attending their placements in a specific setting (e.g., operating theatre), as the data collection tool had been designed and validated to detect UNC in hospital units.

### 2.3. Data Collection Tool and Procedures

The same questionnaire [28] was used in both surveys. The questionnaire consisted of a generic form collecting individual and nursing programme information with 24 closed-ended questions, targeting (a) sociodemographic data (age, gender, civil status and previous work experience); (b) nursing education data (academic year, clinical rotations and places attended); and (c) clinical rotation data concerning the placement attended at the time of the survey (hours/weeks spent in the unit, number of patients cared for and supervision model). Moreover, (d) data concerning the perceived adequacy of resources available in the units in terms of nurses and nursing aides (Likert scale from 1 [*never*], 0% of the time, to 5 [*always*], 100% of the time) and (e) the perceived achievement of the expected learning outcomes (Likert scale from 1 [*not at all*] to 4 [*very greatly*]) were collected.

The questionnaire also included the Unfinished Nursing Care Survey for Students (UNCS4S), validated in Italy [23] and composed in

- Section A, where students were asked to report how often the 22 care interventions were left unfinished or delayed in the last seven days of clinical placement (Likert scale from 1 [*never*] to 5 [*always*]; total score from 22 to 110); and
- Section B, indicating all possible reasons for UNC, listed in 18 items, where students were asked to rank their relevance (Likert scale from 1 [*not a significant reason*] to 5 [*very significant reason*]; total score from 18 to 90).

Data were collected via a Google Forms survey that the Dean of the nursing programme sent after illustrating the study's aims; reminders were sent weekly via email. The online form reported the informed consent on the first page; the whole questionnaire required an average of 15–20 min to be filled in. Students completed the questionnaire during their clinical placement in the last week of attendance, in an appropriate setting (e.g., nurses' room) without any help (e.g., clinical educator).

### 2.4. Statistical Analysis

First, a descriptive analysis was performed to summarise participants' characteristics in the observation period. Averages and confidence intervals (CI 95%) were used for the quantitative variables and frequencies and conditional percentages for factors. The analysis also considers tests on observed differences and chi-squared and *t* tests for quantitative variables and factors, respectively. According to the primary aims of the study, UNCS4S Section A and B averages (CI 95%) were then compared at the item and total score levels using the Wilcoxon test. The overall UNCS4S scores were explored using simple bivariate analyses. Pearson's correlation test was used to discover the relationship between these scores and the continuous variables; correlations were considered weak if lower than 0.30, moderate if lower than 0.70 and strong if greater than 0.70 [29].

The bivariate analyses involving categorical variables were performed by comparing the scores' averages with the ANOVA test (or the equivalent *t* test for dichotomic variables). In addition, according to the secondary aims of the study, given the unidimensionality of the UNCS4S [30], a Mokken scale analysis of UNCS4S Section A was performed to assess differences in the order of nursing interventions left unfinished [29]. The goodness of fit of the model was assessed by computing Loevinger's H coefficient (scalability); the scale was considered weak if 0.30 ≤ H < 0.40, moderate if 0.40 ≤ H < 0.50 and strong if H > 0.50. As reported in Supporting Tables 2 and 3, in the Mokken analysis, both datasets reported a good fit of the model (Loevinger's H coefficient > 0.50). The invariant item ordering was then assessed using HT: Similarly to H, invariant item ordering was considered weak if 0.30 ≤ HT < 0.40, moderate if 0.40 ≤ HT < 0.50 and strong if HT > 0.50 (Supporting Tables 2 and 3).

All analyses were performed using R Core Team [31] in its basic functions and the Mokken library [32, 33]. The statistical significance was set at *p* ≤ 0.05.

### 2.5. Ethical Considerations

The Institutional Review Board of University of Udine approved the study (prepandemic survey: RIF Prot IRB 99/2021; postpandemic survey: RIF Prot IRB 20/2023). Eligible students were homogeneously informed about the aims of the study and its procedures, in both surveys. Then, they were asked to give the informed consent. Students participated freely, without feeling under pressure or having been promised any rewards, apart from the possibility of filling in the questionnaire during the clinical rotation, which was thus considered learning time. The units where students were attending their clinical rotations were informed regarding the ongoing survey. Confidentiality was ensured at the student and at unit and hospital levels.

## 3. Results

### 3.1. Participants

A total of 231 (prepandemic) and 352 (postpandemic group) students participated (Table 1). They were homogeneous in age (average 23.42 vs. 23.49 years, respectively; *p* = 0.878), in gender (86.15% vs. 84.94% female; *p* = 0.718), in academic year attended (mainly first and third; *p* = 0.154) and in the patients cared for (10.50 vs. 11.73; *p* = 0.120), including those newly admitted (2.22 vs. 3.07; *p* = 0.302) (Table 1). However, less previous work experience (30.30% prepandemic vs. 17.90% postpandemic group; *p* = 0.001) and more previous university experience (36.36% vs. 56.25%, respectively; *p* ≤ 0.001) emerged (Table 1). Students had reduced the number of hours per week spent in the unit (35.98 vs. 34.02, respectively; *p* ≤ 0.001), where a significant increase in the adequacy of the staff available was perceived (almost always + always 67.96% vs. 89.20%; respectively *p* ≤ 0.001). Most students reported having achieved their learning outcomes greatly or very greatly, with statistically insignificant differences across groups (69.26% vs. 70.74%; *p* = 0.018) (Table 1).

### 3.2. UNC Interventions and Order

At the overall level, the UNCS4S Section A total score was 39.80 out of 110 (CI 95% 37.06–42.54) in the prepandemic group and 50.89 (CI 95% 47.66–54.12) in the postpandemic group (*p* < 0.001) (Table 2). Two items, namely, spending time with patients and their caregivers (2.33 vs. 2.55, respectively; *p* = 0.084) and informing patients and caregivers on the nursing care they are receiving (2.03 vs. 2.32, respectively; *p* = 0.053), were homogeneous between groups. Among the remaining, significant differences emerged in all cases, indicating an increased perception of UNC among the postpandemic group compared to the prepandemic group (Table 2). The highest difference was reported in performing bedside glucose monitoring as prescribed (1.31 vs. 2.09; *p* < 0.001).

In the bivariate analysis (Supporting Tables 4 and 5), only one individual variable was significantly associated with the total UNCS4S scores among the prepandemic group: the achievement of expected outcomes, greatly to not at all reporting 47.65 vs. 33.84 (*p* = 0.027). In the postpandemic group, two variables were significantly associated with the total UNCS4S score, namely, (1) the academic year attended, with a total score of 52.95 in the first year and 45.53 in the third year (*p* = 0.030) and (2) the adequacy of the staff available in the unit (hardly ever 42.16 vs. 64.17; *p* = 0.003). Moreover, comparing the prepandemic and postpandemic groups, the total UNCS4S average scores were significantly different between those having or not a previous university (40.73 vs. 46.87; *p* = 0.006) or a working experience (47.75 vs. 43.33; *p* = 0.018).

The order in which interventions were left unfinished was completely different (Table 2). For example, monitoring pain as planned was ranked as the 10^th^ intervention left unfinished in the prepandemic group, whereas it was fifth in the postpandemic group.

### 3.3. UNC Reasons

At the overall level, the UNCS4S Section B total scores reported no significant differences between groups (prepandemic 47.91 out of 90, CI 95% 45.10–50.71 vs. postpandemic 45.92, CI 95% 43.91–47.93, *p* = 0.184) (Table 3). At the factor level, a statistical difference emerged in human resources (6.80, CI 95% 6.44–7.15 vs. 6.03, CI 95% 5.77–6.29, respectively; *p* < 0.001) and in workflow predictability (6.18, CI 95% 5.82–6.54 vs. 5.65, CI 95% 5.39–5.91, respectively; *p* = 0.017).

## 4. Discussion

### 4.1. Participants

To the best of our knowledge, this is the first study comparing prepandemic and postpandemic students' perceptions on UNC occurrence and the reasons for them. The main characteristics of participants are in line with those reported in previous studies [16–19, 22]. However, the postpandemic group reported less work experience, likely due to the pandemic, which posed some challenges when searching for work while studying. Meanwhile, they more often reported previous university experience, which may indicate the interruption of studies in other fields due to their attraction to a nursing career as an effect of the pandemic, during which nurses were heroes [34].

Moreover, two main interesting significant differences emerged in the clinical placements: the number of hours spent on a weekly basis, which decreased in the postpandemic group in comparison with the prepandemic one, likely due to the limited learning occasions [27], and the better availability of nursing resources during the postpandemic period, in contrast with the literature [35]. This may be due to the increased number of staff hired during the pandemic and the return to work of nurses after difficult times, when several absences were imposed by COVID-19 infections and suspensions linked to nurses' hesitation to vaccinate. Consequently, students may have perceived staffing as more adequate.

### 4.2. UNC Interventions and Order

Postpandemic students perceived more UNC than prepandemic students in all items and in the overall UNCS4S scores. In fact, postpandemic students reported rarely (average two out of five items) or occasionally (average near to 3) unfinished care, indicating a high occurrence of the phenomenon. These findings suggest a different trend from that documented in available reviews involving nurses [8–11], where a similar pre- and postpandemic occurrence was reported. Differences that emerged are significant and may have both a learning (students) and a clinical (patients) impact, which should be considered with care.

Findings can be interpreted from units' and students' points of view. The occurrence of UNC may have increased because units disrupted their routines due to the changes and transformations imposed by the pandemic: Routines may help keep the main care processes safe [36]. On their side, students may have perceived a high UNC occurrence because they are less equipped to deal with the challenges of practice than in the past, given their limited clinical exposure (e.g., reduced number of hours spent on a weekly basis); moreover, they may be less competent in managing workplace issues given their limited clinical rotations or the acquired fatigue and stress resulting from the pandemic [37]. However, students' individual variables do not influence UNC perceptions, except for previous university and work experiences: The first seems to be protective, while the second seems to increase the perception of UNC, suggesting more investigation of the role of previous experience.

Regarding the order in which the interventions were perceived to be left undone, unlike previous studies documenting homogeneity [38], some new trends emerged. At the Mokken analysis, only two were placed in the same position, indicating the same pre- and postpandemic priority: ensuring clinical teaching of nursing students (in 18^th^ position in both groups) and checking pressure ulcers and changing dressings according to protocols (13^th^ position). Some interventions were placed in similar high- or low-priority positions (between one and three positions before or after); for example,

- High priorities: performing bedside glucose monitoring as prescribed (first in prepandemic and third in postpandemic) and recording vital signs as planned (second and first, respectively).
- Low priorities: supervising tasks assigned to nursing aides (19^th^ and 22^nd^, respectively) and going to patients without being called (20^th^ and 21^st^, respectively).

Other interventions were different in their priority, transiting from high to low or vice versa. For example,

- From high to low priority: spending time with patients and their caregivers (eighth in prepandemic and 19^th^ in postpandemic) and communicating with patients and caregivers (9^th^ and 14^th^, respectively).
- From low to high priority: monitoring pain as planned (10^th^ and fifth, respectively) and monitoring the effects of administered medications (11^th^ and second, respectively).

Therefore, some interventions (e.g., vital signs or glucose monitoring) were ranked as high-priority tasks and were less likely to be left undone than in prepandemic times [8]; others, instead, remained low-priority tasks and at increased risk of being left undone [22].

The prioritisation emerged from students may reflect the priorities seen in their clinical mentors, or their own perspective as students in the first, second or third year of education, thus at different levels of competencies in making independent decisions. Overall, in the postpandemic group, greater attention was paid to patients' basic needs and their clinical evolution by monitoring the administration of medications and their effects and documenting the interventions provided. In contrast, communication and relational support for patients and their caregivers seemed to be sacrificed, while handovers also changed priority (from 6^th^ to 11^th^ position in the order), suggesting that the relationship with colleagues and the continuity of care was seen as less important [39]. While these new priority patterns should be corroborated by additional studies also involving nurses, students should be helped to discuss these issues openly with nurse educators and managers [40] to reflect on underlying reasons and consequences and to promote improvements. The accreditation process of the units as good places for clinical rotations should consider not only the quality of the care delivered but also the nursing staff's readiness to undertake improvement measures.

### 4.3. UNC Reasons

Reasons for UNC, measured by the UNCS4S overall scores, were similar between the two groups: Issues in human resources and communication were considered important as previously documented in available studies [41]. However, some statistical differences emerged only in two factors out of six, namely, in human resources and workflow predictability, both reporting lower averages in the postpandemic group. The shortage of nurses is still an issue after the pandemic [42], while workflow predictability may have been attenuated [7]; however, the practical meaning of the differences that emerged (e.g., in human resources, from 6.80 to 6.03) is limited. In addition, in all items and factors measuring the reasons for UNC, although not significant, smaller averages emerged in the postpandemic compared to the prepandemic group: This may suggest that the increased UNC occurrence perceived by students may be a result of new factors not listed in the UNCS4S tool. A recent systematic review [11] has documented that additional UNC causes emerged during the pandemic according to nurses' perceptions, suggesting that these should be included in the measurement tools to accurately detect all reasons and inform appropriate preventive or mitigating interventions. On the other hand, these findings may suggest that students are not able to detect or hypothesise the reasons for UNC due to some individual factors such as their degree of anxiety or stress [43, 44], exacerbated by the fear of reporting issues occurring in nursing practice.

### 4.4. Strengths and Limitations

To our best knowledge, this is the first study comparing students' UNC perceptions collected before and after the pandemic. However, the monocentric nature of the study may affect the generalisation of the findings. Although students were similar in most individual characteristics, some differences in the pre- and postpandemic surveys may have affected their perceptions. Moreover, despite the good psychometric data of the UNCS4S [23], the findings reflect the perceptions of students and not the real-life UNC occurrence and its reasons.

## 5. Conclusions

There is an increased interest in involving students as a complementary source in UNC investigations as a proxy of the quality of care delivered in the units, especially in comparing the pre- and postpandemic periods, where their *fresh eyes* may help understand the phenomenon's trends and causes. Nursing students' perceptions provide valuable data that enhance our understanding of the challenges and shifts in nursing care practices during these unprecedented times.

Our postpandemic students perceived a higher occurrence of UNC in units attended for their clinical education than prepandemic colleagues. This might show a heightened awareness among students on UNC occurrence or an increased difficulty for them when they face the gap between theory and practice. Moreover, while some interventions are left undone at a similar level to prepandemic data, others have changed their order, suggesting new prioritisation patterns. Future investigations are recommended to accumulate data regarding the changes emerged in the prioritisation, if they actually reflect what students seen in the practice among the nursing staff or their clinical mentors, or if they follow different patterns in deciding priorities, due to for example changes in the education received. Nursing programs sensitive to this phenomenon may play a key role in shaping new patterns of prioritisation. Finally, reasons for UNC perceived by students are in line with those reported in prepandemic times; however, the reduced relevance of some suggests the need for additional research to explain the persistent UNC reasons despite the changes in the healthcare setting findings. Findings may reflect students' difficulties to detect the unfinished care causes as well as the incapacity of the UNCS4S tool developed in the prepandemic times, to capture the postpandemic UNC reasons.

### 5.1. Relevance for Clinical Practice

Taking into consideration their limitations, findings suggest a call for action for both nurse educators and nurse managers. First, patients seem at increased risk of UNC, and students may have been exposed to increased theory–practice gaps in postpandemic times. Appropriate educational strategies to protect their professional development are needed. Second, units should be carefully selected as proper settings for clinical rotations; clinical mentors should be sensitive to the UNC phenomenon, also given the modelling role that they play towards students. Both university educators and clinical mentors should be aware of the UNC phenomenon and help nursing students discuss it from the beginning of their education. Being aware, openly discussing and enacting improvements may mitigate the UNC occurrence and increase the quality of care.

## Figures and Tables

**Table 1:** Characteristics of the participants.

Variable	Prepandemic group, *n* = 231	Postpandemic group, *n* = 352	*p* value
Age (average, CI 95%)	23.42 (22.76, 24.07)	23.49 (22.86, 24.11)	0.878
Gender (*n*, %)			
Female	199 (86.15)	299 (84.94)	
Male	32 (13.85)	53 (15.06)	0.718
Previous university experience (*n*, %)			
Yes	70 (30.30)	63 (17.90)	
No	161 (69.70)	289 (82.10)	0.001
Previous work experience (*n*, %)			
Yes	84 (36.36)	198 (56.25)	< 0.001
No	147 (63.64)	154 (43.75)	
Academic year (*n*, %)			
1st	74 (32.04)	140 (39.77)	
2nd	73 (31.60)	104 (29.55)	
3rd	84 (36.36)	108 (30.68)	0.154
Number of internship hours in the last week (average, CI 95%)	35.98 (35.59, 36.38)	34.02 (33.56, 34.49)	< 0.001
Number of patients cared for in the last shift (average, CI 95%)	10.50 (9.39, 11.60)	11.73 (10.70, 12.76)	0.120
Number of patients newly admitted in the last shift (average, CI 95%)	2.22 (1.86, 2.58)	3.07 (1.77, 4.37)	0.302
Does the unit have an adequate number of nurses in the last training shift? (*n*, %)			
Always (100% of the time)	18 (7.79)	172 (48.86)	
Almost always (75% of the time)	139 (60.17)	142 (40.34)	
Half of the time (50% of the time)	66 (28.57)	30 (8.52)	
Hardly ever (25% of the time)	7 (3.03)	6 (1.70)	
Never (0% of the time)	1 (0.43)	2 (0.57)	< 0.001
Does this clinical rotation allow you to achieve the expected learning outcomes? (Average, CI 95%)			
Very greatly	50 (21.64)	57 (16.19)	
Greatly	110 (47.62)	192 (54.55)	
Enough	65 (28.14)	102 (28.98)	
Not at all	6 (2.60)	1 (0.28)	0.018

Abbreviations: CI, confidence interval; *n*, number.

**Table 2:** Unfinished nursing care as perceived by student's occurrence and order: comparison between pre- and postpandemic groups.

Items, averages (CI 95%)	Prepandemic group	Postpandemic group	*p* value	Order of interventions left unfinished	
				Prepandemic^∗^	Postpandemic^∗^
Go to patients without being called	2.35 (2.16, 2.54)	2.76 (2.61, 2.92)	0.001	20	21
Supervise the tasks assigned to the nurse aides	2.38 (2.19, 2.58)	2.87 (2.70, 3.03)	< 0.001	19	22
Spend time with patients and their caregivers	2.33 (2.14, 2.52)	2.55 (2.39, 2.70)	0.084	8	19
Assess the effectiveness of the care provided, e.g., reviewing if nursing care needs have been met	2.12 (1.93, 2.29)	2.43 (2.26, 2.59)	0.022	3	10
Emotionally support patients and their caregivers	1.95 (1.77, 2.13)	2.56 (2.39, 2.73)	< 0.001	4	7
Communicate with patients and caregivers	2.09 (1.91, 2.27)	2.42 (2.25, 2.60)	0.024	9	14
Teach patients and caregivers how to self-care at home	1.82 (1.64, 2.01)	2.25 (2.09, 2.42)	0.002	14	20
Inform patients and caregivers on nursing care they are receiving	2.03 (1.84, 2.22)	2.32 (2.15, 2.49)	0.053	12	16
Ensure clinical teaching of nursing students	1.90 (1.71, 2.08)	2.28 (2.11, 2.44)	0.006	18	18
Monitor the effects of administered medications	1.72 (1.56, 1.89)	2.33 (2.15, 2.51)	< 0.001	11	2
Document properly the interventions provided and the revision of the care plan	1.79 (1.62, 1.96)	2.23 (2.06, 2.41)	0.005	15	9
Prevent negative outcomes in patients at risk (e.g., falls)	1.70 (1.54, 1.86)	2.27 (2.09, 2.45)	0.001	7	4
Help dependent and/or with dysphagia patients to eat	1.76 (1.59, 1.94)	2.18 (2.00, 2.35)	0.013	21	15
Help dependent and/or with dysphagia patients to drink	1.72 (1.55, 1.88)	2.19 (2.01, 2.37)	0.003	16	6
Administer PRN medications within 15 min of the patient's request	1.62 (1.47, 1.77)	2.23 (2.06, 2.41)	< 0.001	17	12
Prevent healthcare associated infections by adopting good clinical practice (e. g., hand hygiene between patients)	1.61 (1.44, 1.79)	2.20 (2.01, 2.38)	< 0.001	5	8
Check pressure ulcers and change dressing according to protocols	1.60 (1.45, 1.75)	2.26 (2.08, 2.43)	< 0.001	13	13
Monitor pain as planned	1.54 (1.39, 1.69)	2.14 (1.96, 2.32)	< 0.001	10	5
Perform clinical handover to adequately inform the next shift nursing team about patients' conditions	1.49 (1.34, 1.63)	2.07 (1.88, 2.25)	0.002	6	11
Provide personal hygiene to patients who need it	1.61 (1.43, 1.78)	2.18 (2.00, 2.36)	< 0.001	22	17
Record vital signs as planned	1.36 (1.22, 1.49)	2.07 (1.88, 2.26)	< 0.001	2	1
Perform bedside glucose monitoring as prescribed	1.31 (1.18, 1.44)	2.09 (1.89, 2.28)	< 0.001	1	3
Total score UNCS4S (from 22 to 110)	39.80 (37.06, 42.54)	50.89 (47.66, 54.12)	< 0.001		

*Note:* Item score: from 1 (*never unfinished*) to 5 (*always unfinished*). Total score: from 22 (*never unfinished*) to 110 (*always unfinished*).

Abbreviations: CI, confidence interval; PRN, pro re nata; UNCS4S, Unfinished Nursing Care survey for Students.

^∗^See Supporting Tables 2 and 3.

**Table 3:** Reasons of unfinished nursing care as perceived by students: comparison between pre- and postpandemic groups.

Items, factors	Prepandemic group	Postpandemic group	*p* value
	Average (CI 95%)	Average (CI 95%)	
Factor 1: communication (from 5 to 25)	13.20 (12.30, 14.09)	12.57 (11.95, 13.20)	0.537
Tension/conflicts within the nursing staff	2.52 (2.33, 2.71)	2.50 (2.33, 2.66)	0.319
Incomplete or interrupted communication among nursing staff	2.66 (2.47, 2.86)	2.58 (2.43, 2.72)	0.325
Tension/conflicts between nursing and medical staff	2.63 (2.44, 2.83)	2.44 (2.28, 2.60)	0.057
Incomplete/interrupted communication between nursing and medical staff	2.74 (2.54, 2.94)	2.67 (2.53, 2.82)	0.544
Lack of support/collaboration among team members	2.63 (2.43, 2.83)	2.39 (2.24, 2.53)	0.067

Factor 2: priority setting (from 5 to 15)	7.28 (6.79, 7.76)	7.01 (6.60, 7.41)	0.332
Inadequate nursing care model (e.g., task-oriented model of care)	2.51 (2.33, 2.69)	2.42 (2.27, 2.57)	0.217
Inaccurate initial priority setting	2.35 (2.16, 2.53)	2.29 (2.13, 2.44)	0.257
Inadequate priority reassessment during the shift	2.45 (2.27, 2.62)	2.29 (2.14, 2.45)	0.022

Factor 3: Nurses' aides' supervision (from 5 to 15)	7.15 (6.66, 7.64)	7.16 (6.74, 7.57)	0.926
Nurse aides missed or delayed the reporting of the tasks left undone	2.33 (2.15, 2.50)	2.33 (2.18, 2.48)	0.376
Inadequate supervision of the tasks assigned to the nurse aides	2.32 (2.14, 2.50)	2.40 (2.24, 2.55)	0.865
Incomplete or interrupted communication between nursing staff and nurse aides/assistive personnel	2.50 (2.31, 2.68)	2.43 (2.28, 2.57)	0.362

Factor 4: material resources (from 5 to 15)	7.72 (7.24, 8.20)	7.50 (7.12, 7.88)	0.525
Medications prescribed not available	2.65 (2.46, 2.84)	2.48 (2.34, 2.62)	0.130
Equipment not available/not functioning properly when needed	2.57 (2.39, 2.75)	2.44 (2.29, 2.59)	0.193
Other departments did not provide the service expected (e.g., delay in diagnostic processes)	2.47 (2.30, 2.64)	2.58 (2.44, 2.72)	0.817

Factor 5: human resources (from 5 to 10)	6.80 (6.44,7.15)	6.03 (5.77, 6.29)	< 0.001
Inadequate number of nurses	3.54 (3.35, 3.73)	3.05 (2.92 3.19)	< 0.001
Inadequate number of staff support	3.23 (3.04, 3.43)	2.98 (2.84, 3.11)	0.081

Factor 6: workflow predictability (from 5 to 10)	6.18 (5.82, 6.54)	5.65 (5.39, 5.91)	0.017
Unexpected increase in the number of patients in critical conditions	3.10 (2.91, 3.30)	2.80 (2.66, 2.95)	0.022
High number of hospitalisations/discharges during the shift	3.07 (2.87, 3.27)	2.85 (2.70, 2.99)	0.104

Total score UNCS4S (from 18 to 90)	**47.91 (45.10, 50.71)**	**45.92 (43.91, 47.93)**	**0.184**

*Note:* Item score: from 1 (*not a significant reason*) to 5 (*very significant reason*). Total score: from 18 (*not a significant reason*) to 90 (*very significant reason*).

Abbreviations: CI, confidence interval; UNCS4S, Unfinished Nursing Care Survey for Students.

## Data Availability

Data are available at the request from the authors.

## Supporting Information

Supporting Information 1 Supporting Table 1: Strengthening the Reporting of Observational Studies in Epidemiology Statement for observational studies.

Supporting Information 2 Supporting Table 2: Unfinished Nursing Care Survey for Students in prepandemic, Section A: results of Mokken Scale.

Supporting Information 3 Supporting Table 3: Unfinished Nursing Care Survey for Students in postpandemic, Section A: results of Mokken Scale.

Supporting Information 4 Supporting Table 4: Unfinished Nursing Care Survey for Students average scores Section A (22 items) and demographic characteristics: bivariate analysis.

Supporting Information 5 Supporting Table 5: Unfinished Nursing Care Survey for Students Section A (22 items) and correlations with demographic characteristics as continuous variables.
