# Supplementary material for: Changes in the Pre- and Postpandemic Unfinished Nursing Care Occurrence and Reasons as Perceived by Italian Nursing Students: A Secondary Analysis
Source: J Nurs Manag. 2025 Apr 7;2025:8892363. doi: 10.1155/jonm/8892363 (PMC11996277; doi:10.1155/jonm/8892363)
Supplement: Supporting Information 2 — Supporting Table 2: Unfinished Nursing Care Survey for Students in prepandemic, Section A: results of Mokken Scale. [file 8892363.f2.docx]

**SUPPLEMENTARY TABLE 2 |** Unfinished Nursing Care Survey for Students in pre-pandemic, Section A: results of Mokken Scale.

| **Item** | **H** | **SE** | **Mon_crit** | **item_ord_crit.tsig** | **item_ord_crit.crit** | **item_sel** |
| --- | --- | --- | --- | --- | --- | --- |
| Provide personal hygiene to patients who need it | 0.462 | (0.082) | 0 | 0 | 27 | 1 |
| Help dependent and/or with dysphagia patients to eat | 0.519 | (0.063) | 0 | 1 | 45 | 0 |
| Go to patients without being called | 0.520 | (0.051) | 0 | 0 | 26 | 1 |
| Supervise the tasks assigned to the nurse aides | 0.528 | (0.054) | 0 | 0 | 0 | 1 |
| Ensure clinical teaching of nursing students | 0.539 | (0.062) | 0 | 0 | 0 | 1 |
| Administer PRN* medications within 15 min of the patient’s request | 0.555 | (0.066) | 0 | 1 | 52 | 1 |
| Help dependent and/or with dysphagia patients to drink | 0.557 | (0.061) | 0 | 1 | 40 | 1 |
| Document properly the interventions provided and the revision of the care plan | 0.559 | (0.059) | 0 | 1 | 55 | 1 |
| Teach patients and caregivers how to self-care at home | 0.560 | (0.055) | 0 | 0 | 0 | 1 |
| Check pressure ulcers and change dressing according to protocols | 0.561 | (0.063) | 0 | 0 | 0 | 1 |
| Inform patients and their caregivers about nursing care they are receiving | 0.593 | (0.047) | 0 | 0 | 0 | 1 |
| Monitor the effects of administered medications | 0.600 | (0.054) | 0 | 2 | 61 | 0 |
| Monitor pain as planned | 0.608 | (0.061) | 0 | 0 | 0 | 1 |
| Communicate with patients and caregivers | 0.620 | (0.044) | 0 | 0 | 0 | 1 |
| Spend time with patients and their caregivers | 0.627 | (0.044) | 0 | 0 | 21 | 1 |
| Prevent negative outcomes for patients at risk (e.g. falls, pressure ulcers, malnutrition) | 0.628 | (0.051) | 0 | 0 | 0 | 1 |
| Perform clinical handover to adequately inform the next shift nursing team about patients’ conditions | 0.629 | (0.058) | 0 | 0 | 0 | 1 |
| Prevent healthcare associated infections by adopting good clinical practice (e. g. hand hygiene between patients, closed urinary drainage system) | 0.635 | (0.050) | 0 | 0 | 18 | 1 |
| Emotionally support patients and their caregivers | 0.639 | (0.044) | 0 | 0 | 0 | 1 |
| Assess the effectiveness of the care provided, e.g. reviewing if nursing care needs have been met | 0.640 | (0.044) | 0 | 0 | 0 | 1 |
| Record vital signs as planned | 0.650 | (0.068) | 0 | 0 | 0 | 1 |
| Perform bedside glucose monitoring as prescribed | 0.660 | (0.069) | 0 | 0 | 0 | 1 |
| **Total** | **0.583** | **(0.048)** |  |  |  | **1** |

**Abbreviations:** H, scalability index; SE, Standard Error.
