# Supplementary material for: Changes in the Pre- and Postpandemic Unfinished Nursing Care Occurrence and Reasons as Perceived by Italian Nursing Students: A Secondary Analysis
Source: J Nurs Manag. 2025 Apr 7;2025:8892363. doi: 10.1155/jonm/8892363 (PMC11996277; doi:10.1155/jonm/8892363)
Supplement: Supporting Information 3 — Supporting Table 3: Unfinished Nursing Care Survey for Students in postpandemic, Section A: results of Mokken Scale. [file 8892363.f3.docx]

**SUPPLEMENTARY TABLE 3 |** Unfinished Nursing Care Survey for Students in post-pandemic, Section A: results of Mokken Scale.

| **Item** | **H** | **SE** | **Mon_crit** | **item_ord_crit.tsig** | **item_ord_crit.crit** | **item_sel** |
| --- | --- | --- | --- | --- | --- | --- |
| Supervise the tasks assigned to the nurse aides | 0.448 | (0.052) | 18 | 4 | 192 | 0 |
| Go to patients without being called | 0.614 | (0.042) | 0 | 1 | 39 | 0 |
| Teach patients and caregivers how to self-care at home | 0.681 | (0.034) | 0 | 1 | 63 | 1 |
| Spend time with patients and their caregivers | 0.716 | (0.031) | 0 | 1 | 48 | 0 |
| Ensure clinical teaching of nursing students | 0.726 | (0.029) | 0 | 0 | 12 | 1 |
| Provide personal hygiene to patients who need it | 0.730 | (0.029) | 0 | 2 | 104 | 0 |
| Inform patients and their caregivers about nursing care they are receiving | 0.733 | (0.027) | 0 | 0 | 21 | 1 |
| Help dependent and/or with dysphagia patients to eat | 0.739 | (0.028) | 0 | 0 | 21 | 1 |
| Communicate with patients and caregivers | 0.740 | (0.028) | 0 | 0 | 0 | 1 |
| Check pressure ulcers and change dressing according to protocols | 0.746 | (0.027) | 0 | 0 | 28 | 1 |
| Administer PRN* medications within 15 min of the patient’s request | 0.748 | (0.025) | 0 | 2 | 85 | 0 |
| Perform clinical handover to adequately inform the next shift nursing team about patients’ conditions | 0.763 | (0.027) | 0 | 1 | 55 | 1 |
| Assess the effectiveness of the care provided, e.g. reviewing if nursing care needs have been met | 0.764 | (0.023) | 0 | 0 | 0 | 1 |
| Document properly the interventions provided and the revision of the care plan | 0.765 | (0.024) | 0 | 1 | 30 | 1 |
| Prevent healthcare associated infections by adopting good clinical practice (e. g. hand hygiene between patients, closed urinary drainage system) | 0.767 | (0.025) | 0 | 0 | 43 | 1 |
| Emotionally support patients and their caregivers | 0.771 | (0.023) | 0 | 1 | 45 | 1 |
| Help dependent and/or with dysphagia patients to drink | 0.777 | (0.024) | 0 | 1 | 37 | 1 |
| Monitor pain as planned | 0.780 | (0.024) | 0 | 0 | 0 | 1 |
| Prevent negative outcomes for patients at risk (e.g. falls, pressure ulcers, malnutrition) | 0.788 | (0.021) | 0 | 0 | 0 | 1 |
| Perform bedside glucose monitoring as prescribed | 0.788 | (0.026) | 0 | 0 | 0 | 1 |
| Monitor the effects of administered medications | 0.792 | (0.020) | 0 | 2 | 127 | 1 |
| Record vital signs as planned | 0.814 | (0.020) | 0 | 1 | 90 | 1 |
| **Total** | **0.739** | **(0.021)** |  |  |  | **1** |

**Abbreviations:** H, scalability index; SE, Standard Error.
