# Supplementary material for: Changes in the Pre- and Postpandemic Unfinished Nursing Care Occurrence and Reasons as Perceived by Italian Nursing Students: A Secondary Analysis
Source: J Nurs Manag. 2025 Apr 7;2025:8892363. doi: 10.1155/jonm/8892363 (PMC11996277; doi:10.1155/jonm/8892363)
Supplement: Supporting Information 4 — Supporting Table 4: Unfinished Nursing Care Survey for Students average scores Section A (22 items) and demographic characteristics: bivariate analysis. [file 8892363.f4.docx]

**SUPPLEMENTARY TABLE 4 |** Unfinished Nursing Care Survey for Students average scores Section A (22 items) and demographic characteristics: bivariate analysis.

| **Variables** | **Overall**  **(n=583)** | **Pre-pandemic group**  **(n=231)** | **Post-pandemic group**  **(n=352)** |
| --- | --- | --- | --- |
| Gender, average (CI 95%)  Female  Male  p-value | 45.67 (43.67; 47.68)  44.26 (39.78; 48.73)  0.592 | 39.53 (36.91; 42.16)  37.49 (33.17; 41.81)  0.552 | 49.76 (46.99; 52.53)  48.34 (41.80; 54.89)  0.695 |
| Previous University Experience, average (CI 95%)  Yes  No  p-value | 40.73 (37.44; 44.02)  46.87 (44.72; 49.02)  0.006 | 37.59 (34.08; 41.11)  39.97 (36.98; 42.96)  0.356 | 44.21 (38.48; 49.94)  50.71 (47.89; 53.54)  0.053 |
| Previous Work Experience, average (CI 95%)  Yes  No  p-value | 47.75 (44.96; 50.54)  43.33 (40.95; 45.72)  0.018 | 39.27 (35.61; 42.94)  39.24 (36.20; 42.27)  0.987 | 51.34 (47.78; 54.90)  47.24 (43.67; 50.82)  0.116 |
| Academic year attended, average (CI 95%)  I  II  III  p-value | 48.28 (45.04, 51.52)  42.91 (39.71, 46.11)  44.69 (41.65, 47.72)  0.053 | 39.45 (35.38, 43.53)  40.41 (35.99, 44.83)  38.06 (34.25, 41.88)  0.713 | 52.95 (48.65, 57.24)  44.67 (40.16, 49.17)  49.84 (45.53,54.15)  0.030 |
| Does the unit have an adequate number of nurses in the last training shift?, average (CI 95%)  Always (100% of the time)  Almost always (75% of the time)  Half of the time (50% of the time)  Hardly ever (25% of the time)  Never (0% of the time)  p-value | 44.01 (40.41, 47.60)  46.24 (43.72, 48.76)  44.81 (40.79, 48.83)  52.32 (38.44, 66.20)  57.20 (4.93, 109.48)  0.521 | 39.09 (26.62, 51.57)  38.65 (35.77, 41.53)  40.23 (35.91, 55.56)  42.16 (23.14, 61.18)  40.00 (NA)  0.970 | 44.52 (40.73, 48.31)  53.66 (49.90, 57.43)  54.88 (47.00, 62.76)  64.17 (42.01, 86.32)  65.81 (-123.00, 254.62)  0.003 |
| Has this clinical rotation allowed you to achieve the expected learning outcomes?, average (CI 95%)  Not at all  Enough  Greatly  Very Greatly  p-value | 49.99 (29.94, 70.03)  48.02 (44.83, 51.21)  45.62 (43.05, 48.19)  40.75 (36.15, 45.35)  0.067 | 47.65 (23.77, 71.53)  43.23 (38.67, 47.80)  38.90 (35.66, 42.13)  33.84 (28.85, 38.84)  0.027 | 64.00 (NA)  51.07 (46.77, 55.37)  49.48 (45.98, 52.97)  46.80 (39.59, 54.02)  0.685 |

**Abbreviations:** %, percentage; CI, Confidence Interval; n, numbers; NA, Not appropriate given the amount of nursing students with that characteristics; UNCS4S, Unfinished Nursing Care Survey for Students.
