# Supplementary material for: Changes in the Pre- and Postpandemic Unfinished Nursing Care Occurrence and Reasons as Perceived by Italian Nursing Students: A Secondary Analysis
Source: J Nurs Manag. 2025 Apr 7;2025:8892363. doi: 10.1155/jonm/8892363 (PMC11996277; doi:10.1155/jonm/8892363)
Supplement: Supporting Information 5 — Supporting Table 5: Unfinished Nursing Care Survey for Students Section A (22 items) and correlations with demographic characteristics as continuous variables. [file 8892363.f5.docx]

**SUPPLEMENTARY TABLE 5 |.** Unfinished Nursing Care Survey for Students Section A (22 items) and correlations with demographic characteristics as continuous variables.

| **Variables** | **Overall**  **(n=583)** | **Pre-pandemic**  **group**  **(n=231)** | **Post-pandemic group**  **(n=352)** |
| --- | --- | --- | --- |
| Age  p-value | 0.075 (-0.007; 0.155)  0.072 | 0.076 (-0.054; 0.203)  0.252 | 0.075 (-0.030; 0.178)  0.161 |
| Number of hours per internship  p-value | -0.029 (-0.110; 0.052)  0.481 | -0.042 (-0.170; 0.087)  0.525 | 0.048 (-0.057; 0.152)  0.371 |
| Number of patients cared for in the last shift of clinical training  p-value | 0.043 (-0.038; 0.124)  0.301 | -0.019 (-0.148; 0.111)  0.776 | 0.049 (-0.056; 0.153)  0.358 |
| Number of patients newly admitted in the last shift of clinical training  p-value | 0.076 (-0.005; 0.157)  0.067 | 0.052 (-0.077; 0.180)  0.430 | 0.076 (-0.030; 0.179)  0.159 |

**Abbreviations:** n, number.
